# Prospective analysis of different combined regimens of stereotactic body radiation therapy and chemotherapy for locally advanced pancreatic cancer

**DOI:** 10.1002/cam4.1553

**Published:** 2018-05-17

**Authors:** Xiaofei Zhu, Dongchen Shi, Fuqi Li, Xiaoping Ju, Yangsen Cao, Yuxin Shen, Fei Cao, Shuiwang Qing, Fang Fang, Zhen Jia, Huojun Zhang

**Affiliations:** ^1^ Department of Radiation Oncology Changhai Hospital Affiliated to Navy Medical University Shanghai China; ^2^ Department of Pulmonary and Critical Care Medicine Changhai Hospital Affiliated to Navy Medical University Shanghai China

**Keywords:** chemotherapy, locally advanced pancreatic cancer, multimodality, stereotactic body radiation therapy, treatment strategy

## Abstract

To identify impacts of different combined regimens of stereotactic body radiation therapy (SBRT) and chemotherapy on survival of patients with locally advanced pancreatic cancer (LAPC) and factors correlated with determinations of different combinations. Four hundred and nineteen patients with radiographically and biopsy‐proven LAPC were prospectively enrolled. Factors associated with different strategies were analyzed with Chi‐square test and contingency coefficients. Cox regression was used to identify factors predictive of survival. Prognostic values of different multimodality were further analyzed by propensity score‐matched analysis. Median overall survival (OS) and progression‐free survival (PFS) of all patients was 13.2 and 8.2 months, respectively. Baseline ECOG correlated with induction chemotherapy, while tumor stage, lymph node invasion, and toxicity after SBRT associated with adjuvant chemotherapy. Patients with induction chemotherapy alone (12.2 months), adjuvant chemotherapy alone (13.6 months), and induction and adjuvant chemotherapy (13.3 months) had longer OS than those without chemotherapy (11.2 months; *P* < .001), while adjuvant chemotherapy alone and induction and adjuvant chemotherapy increased PFS. An adjusted overall survival benefit was gained with adjuvant chemotherapy compared with induction and adjuvant chemotherapy (OS: 14.7 months [95% CI: 14.2‐15.2 months] vs 13.1 months [95% CI: 12.3‐13.9 months]; *P* < .001) (PFS: 8.8 months [95% CI: 8.4‐9.2 months] vs 8.1 months [95% CI: 7.4‐8.8 months]; *P* = .053). Induction and adjuvant chemotherapy, especially adjuvant chemotherapy, plus SBRT may improve OS and PFS. Baseline performance status, tumor stage, lymph node involvement, and toxicity after SBRT influenced determinations of upfront multimodality.

## 1 INTRODUCTION

Pancreatic cancer has been the fourth leading cause of cancer mortality in the USA with a dismal 5‐year survival rate of 8%.^1^ The latest findings also showed that in contrast to the declining trends for the 4 major cancers, the mortality of pancreatic cancer continues to increase slightly (by 0.3% per year) in men but have leveled off in women.^2^ Similar trends were found in China with increasing incidences and cancer deaths.^3^

Due to its insidious symptoms and unsuccessful population‐based screenings, majority of patients had locally advanced pancreatic cancer (LAPC) at the initial diagnosis. In spite of potential survival benefit over radiotherapy or chemotherapy alone produced by concurrent chemoradiotherapy,^4^, ^5^, ^6^, ^7^, ^8^ contrary conclusions were also clarified.^9^, ^10^, ^11^, ^12^

As a result, there is no consensus on the optimal management of LAPC. Hence, chemotherapy or induction chemotherapy followed by chemoradiation or chemoradiation alone or enrollment of clinical trials was employed based on NCCN guidelines. While given the shortcomings of conventional radiotherapy, stereotactic body radiation therapy (SBRT) has been a promising option in pancreatic cancer due to its inherent advantages including high local dose conformation, precise target localization 13 with motion compensation strategies 14, 15 and quick dose fall‐off outside the tumor volume.^16^, ^17^

Although chemotherapy and radiotherapy have played a pivotal role in the treatment of patients with LAPC, several controversial issues remain unresolved. Particularly, the best upfront combined regimens of different modalities as well as the optimal treatment strategy are still a matter of debate.

Therefore, in this study, we sought to evaluate impacts of different multimodality for LAPC on overall survival (OS) and progression‐free survival (PFS) and factors correlated with determinations of treatment strategies.

## 2 METHODS

The study was approved by the institutional review board of our hospital. Data were collected prospectively from 2012 to 2016. All patients were carefully assessed before treatment based on the medical records, imaging studies, histological examinations and laboratory tests. A prospective maintained pancreatic cancer database was used to identify consecutive patients who had LAPC and received SBRT between January 2012 and December 2016. Informed consents, including publication of details, of all patients were provided before treatment.

### 2.1 Eligibility

All patients included in this study were LAPC. Patients were eligible for inclusion if meeting the following criteria: (1) biopsy‐proven and radiologically locally advanced pancreatic cancer, (2) ECOG performance status ≤2, (3) leukocyte count ≥3.5 × 10^9^/L, neutrophil count ≥1.5 × 10^9^/L, hemoglobin level ≥100 g/L, platelet count ≥100 × 10^9^/L and normal liver and kidney function, and (4) completion of a planned chemotherapy with 6 cycles.

Patients who had completed induction chemotherapy would receive PET‐CT to preclude metastasis. Those with metastasis were excluded from the study and received other treatment based on the multidisciplinary approach. Those without metastasis would receive SBRT thereafter.

### 2.2 Staging

Comprehensive clinical and radiographic staging, including chest computed tomography and abdominal computed tomography or magnetic resonance imaging scan and laboratory studies, was mandatory prior to treatment. Furthermore, pathological examinations with fine‐needle aspiration guided by endoscopic ultrasound were preferred for all patients. The most recent results of laboratory and imaging studies before initiation of treatment were utilized for analysis. Consensus regarding the definition of LAPC was provided by the multidisciplinary team based on NCCN guidelines.

### 2.3 Chemotherapy

Patients were required to receive gemcitabine and S‐1 in addition to SBRT. However, other palliative care would be given if patients were intolerant of chemotherapy. S‐1 was the prodrug of 5‐fluorouracil comprising of tegafur, gimeracil, and oteracil. It was proven that S‐1 was not inferior to gemcitabine in terms of overall survival rates and progression‐free survival rates with tolerable effects.^18^, ^19^, ^20^, ^21^ Patients were recommended to receive up to 6 months of chemotherapy. The interval between SBRT and chemotherapy was 2‐3 weeks. Intravenous administration of gemcitabine (1000 mg/m^2^) was initiated on days 1, 8, and 15 during each 4‐week cycle, which repeated for 6 cycles. S‐1 was orally given at a dose of 80 mg/m^2^ for 28 days followed by a 14‐day rest, which also continued for 6 cycles.

### 2.4 Treatment planning and delivery

The protocol of SBRT was similar to our previous studies.^22^, ^23^ SBRT was delivered *via* CyberKnife^®^ (Accuray Incorporated, Sunnyvale, CA), an image‐guided frameless stereotactic robotic radiosurgery system. All patients underwent endoscopic ultrasound‐guided implantation of 3‐5 gold fiducials within or adjacent to the pancreatic tumor. Patients underwent CT simulation supine in custom‐fit immobilization devices with intravenous contrast. Gross tumor volume (GTV) was delineated as a radiographically evident gross disease by contrast CT. Clinical target volume (CTV) encompassing areas of the potential subclinical disease spread was also designated. In most cases, the CTV equaled GTV. Planning target volume (PTV) included a 2‐5‐mm margin on GTV. When the tumor abutted critical organs, the expansion of PTV outside of CTV in this direction should be avoided. Therefore, the margin expansion was allowed to be nonuniform. At least ninety percent of PTV should be covered by the prescription dose. Normal tissue constraints were referred to the American Association of Physicists in Medicine guidelines in TG‐101.^24^

### 2.5 Follow‐up

Patients were evaluated initially every 2‐3 months within 1 year after treatment and later every 4‐6 months with CT or MRI scans, physical examinations, and CA19‐9 for a planned follow‐up of 5 years. Any other examinations prompted by new‐onset symptoms or at the physician’s discretions were also used to record events.

### 2.6 Definitions and collection of data

Disease recurrence was based on review of the medical records and imaging studies, including newly found mass or growth of the primary lesion. A new low‐density mass on CT or MRI consistent with recurrent local, regional, or metastatic disease was considered as such, and tumor biopsy was rarely performed.^25^ Differential diagnosis of tumor necrosis induced by SBRT, which may be mistaken for progression, would be performed by three radiologists based on MRI scan. OS was determined from the date of histologic diagnosis to death. The definition of PFS was from the date of histologic diagnosis to the date of the first recurrence. Tumor response was judged by RECIST Criteria version 1.1. Adverse effects caused by chemotherapy were reviewed and collected by Common Terminology Criteria for Adverse Events (CTCAE) Version 4.0. Radiation‐induced acute toxicities were determined by “Acute radiation morbidity scoring criteria” from Radiation Therapy Oncology Group. While late toxicities were evaluated by “Late radiation morbidity scoring schema” from Radiation Therapy Oncology Group/European Organization for Research on the Treatment of Cancer.^26^

A systemic inflammation response index (SIRI) has been proven predictive of prognosis of patients with pancreatic cancer.^27^ The value was calculated as:

$$\begin{array}{cc} \text{SIRI} & =\\ & \frac{\text{total neutrophil count}\;\left(/{\text{mm}}^{3}\right)\;\times \;\text{total monocyte count}\;\left(/{\text{mm}}^{3}\right)}{\text{total lymphocyte count}\;(/{\text{mm}}^{3})}. \end{array}$$

The prognostic nutritional index (PNI) represented patient’s nutritional status, which was the known predictor of the survival of pancreatic cancer.^28^, ^29^ The formula was as follows: PNI = 10 × serum albumin (g/dL) + 0.005 × total lymphocyte count (/mm^3^). Charlson age‐comorbidity index (CACI) was originally designed to classify prognostic comorbidity.^30^ It was clarified that CACI was associated with prognosis of patients with pancreatic cancer.^31^ Pain was quantified by visual analog scale (VAS).

The recommended upper limit of normal for CA19‐9 is 37 U/mL.^32^ Additionally, a phase I/II study of *nab‐*Paclitaxel + Gemcitabine that preceded advanced pancreatic cancer demonstrated a significant correlation between decreases in CA19‐9 levels of ≥50% vs <50% from baseline and improved survival.^33^ Therefore, CA19‐9 response was defined as the level of CA19‐9 decrease by 50% from baseline levels of ≥74 U/mL. Hence, three CA19‐9 groups were formed for univariate analysis: CA19‐9 levels ≥74 U/mLwith response vs CA19‐9 levels ≥74 U/mL with no response (including CA19‐9 levels within the normal range before SBRT while increased after SBRT) vs CA19‐9 levels <74 U/mL (before SBRT and during follow‐up). The nadir value of CA19‐9 level during the follow‐up was utilized for the estimation of CA19‐9 decrease. Additionally, it was shown that CA19‐9 level less than 200 U/mL was associated with major response for localized pancreatic cancer treated with preoperative therapy.^34^ Therefore, the serum level of CA19‐9 before SBRT was stratified as: <200 and ≥200 U/mL.

The N stage (TNM staging) was based on the absence or presence of metastasis to the regional lymph nodes, which located along the drainage pathway that were included in the surgical field.^35^ The presence of lymph node invasion in imaging was defined as short axis >1 cm, abnormal round morphology, heterogeneity shown in imaging, or central necrosis.^35^

### 2.7 Propensity score matching

To create 2 matching groups of patients with different combinations of treatment modality, a logistic regression model was built with the modality as the dependent variable and all other variables that could potentially influence its prognostic impact as independent variables. These variables were shown to correlate with survival after multivariate regression analysis.

### 2.8 Statistical analysis

Baseline characteristics were summarized by descriptive statistics. Factors associated with determinations of multimodality were analyzed with Chi‐square test and its contingency coefficient (*C*). Potential factors predictive of OS and PFS were identified with univariate log‐rank comparisons and then multivariate proportional hazards regression model. Survival probability was estimated using Kaplan‐Meier statistics. Impacts of different treatment strategies on survival were evaluated with propensity score‐matched analysis. Two‐sided *P* values <.05 were considered statistically significant. Statistical analyses were performed using IBM SPSS version 22.0 (SPSS Inc., Armonk, NY).

## 3 RESULTS

### 3.1 Demographics

Four hundred and nineteen patients with LAPC were included. Demographic characteristics are outlined in Table 1 in a detailed manner. Median follow‐up was 13 months (range: 5‐33 months). The median prescription dose and BED_10_ (biological effective dose, α/β=10) were 36 Gy (range: 30‐49.6 Gy) and 61.92 Gy (range: 48‐85.5 Gy) in 5‐8 fractions, respectively.

**Table 1:** Baseline patient characteristics

Characteristics	Value
All patients	419
Sex	
Male	250
Female	169
Age (y)	66 y (29‐90 y)
ECOG PS	
0	120 (28.6%)
1	216 (51.6%)
2	83 (19.8%)
Stage	
IIA	76 (18.1%)
IIB	210 (50.1%)
III	133 (31.7%)
Tumor locations	
Head	283 (67.5%)
Body and tail	136 (32.5%)
Tumor diameter (cm)	3.98 (2.4‐9.0)
Baseline CA19‐9 (U/mL)	
<200	163 (38.9%)
≥200	256 (61.1%)
Treatment sequence	
Nonchemotherapy	33 (7.9%)
Induction chemotherapy alone	45 (10.7%)
Adjuvant chemotherapy alone	205 (48.9%)
Induction and adjuvant chemotherapy	136 (32.5%)
Prescription dose	30‐49.6 Gy/5‐8f
BED_10_	61.92 Gy (48‐85.5 Gy)

BED_10_, biological effective dose, α/β=10.

### 3.2 Factors correlated with determinations of different treatment modality

Induction chemotherapy (including with or without adjuvant chemotherapy) was performed in 181 patients. A significant association was found only between baseline ECOG and induction chemotherapy (*C* = 0.582, *P* < .001). Additionally, more patients with ECOG of 0 point (n = 119, 99.2%) had induction chemotherapy than those with ECOG of 1 or 2 points (n = 62, 20.7%; *P* < .001). A total of 341 patients had adjuvant chemotherapy (including with or without induction chemotherapy). Tumor stage (*C* = 0.644, *P* < .001), tumor diameter (*C* = 0.350, *P* < .001), lymph node invasion (*C* = 0.467, *P* < .001), and radiation‐induced toxicities within 2‐3 weeks after SBRT (*C* = 0.687, *P* < .001) correlated with adjuvant chemotherapy. In details, adjuvant chemotherapy was performed more frequently in patients with stage III (n = 131, 98.5%; *P* < .001) and lymph node invasion (n = 258, 97.0%; *P* < .001) than those with stage II (n = 210, 73.4%) and noninvolvement of lymph nodes (n = 83, 54.2%). In addition, more patients with no radiation‐induced toxicities within 2‐3 weeks after SBRT (better performance status) underwent chemotherapy as scheduled (n = 273, 100%) than those with toxicities (n = 68, 46.6%; *P* < .001).

### 3.3 Factors predictive of OS

Three hundred and sixty‐five patients died, while only 54 patients were still alive at the last follow‐up. The median OS was 13.2 months (95% CI: 12.8‐13.6 months). Furthermore, 1‐year and 2‐year OS rate was 63.0% and 13.6%, respectively. Before treatment, a level of CA19‐9 ≥ 200 U/mL was found in 257 patients, while 162 patients had a level <200 U/mL. Among patients with a level of CA19‐9 ≥ 2 upper limit of normal, a significant decrease was found in 193 patients, while 134 patients had no response or even elevated levels of CA19‐9 after treatment. On the univariate analysis, age, different combinations of treatment modality, SIRI, CACI, CA19‐9 level, CA19‐9 response and BED_10_ were predictive of OS (Table 2). On multivariate regression, age, different combinations of treatment modality, CA19‐9 response and BED_10_ correlated with OS (Table 2).

**Table 2:** Univariate and multivariate analysis of factors associated with OS

Variable	N = 419	Univariate, overall survival (mo)		*P* value (log‐rank)	Multivariate, hazard ratio			*P* value (Cox regression)
		Median	95% CI		HR	95% CI	*B*	
Age								
<65	191	14.0	13.3‐14.6	.021	1			.025
≥65	228	12.8	12.2‐13.4		1.29	1.03‐1.62	0.26	
Smoking								
Absent	302	13.1	12.5‐13.7	.564	NS	NS	NS	NS
Present	117	13.6	12.8‐14.4		NS	NS	NS	
Diabetes mellitus								
Absent	322	13.4	13.0‐13.8	.320	NS	NS	NS	NS
Present	95	12.5	11.9‐13.1		NS	NS	NS	
VAS								
<3	158	12.5	11.5‐13.5	.455	NS	NS	NS	NS
≥3	261	13.5	13.1‐13.9		NS	NS	NS	
Weight loss								
<5 kg	221	13.1	12.4‐13.8	.306	NS	NS	NS	NS
≥5 kg	198	13.3	12.8‐13.8		NS	NS	NS	
Tumor diameter								
<4 cm	227	13.2	12.7‐13.6	.726	NS	NS	NS	NS
≥4 cm	192	13.2	12.6‐13.8		NS	NS	NS	
ECOG								
0	120	13.2	11.7‐14.7	.159	NS	NS	NS	
1	216	13.1	12.4‐13.8		NS	NS	NS	NS
2	83	13.7	12.7‐14.7		NS	NS	NS	
Treatment modality								
Nonchemotherapy	33	11.2	10.5‐11.8	<.001	1			<.001
Induction chemotherapy	45	12.2	11.3‐13.0		0.60	0.37‐0.99	−0.51	.046
Adjuvant chemotherapy	205	13.6	13.0‐14.1		0.42	0.28‐0.62	−0.88	<.001
Induction and adjuvant chemotherapy	136	13.3	12.4‐14.2		0.50	0.33‐0.76	−0.69	.001
SIRI								
<1.0	230	13.9	13.3‐14.5	.012	NS	NS	NS	NS
≥1.0	189	12.4	11.8‐13.0		NS	NS	NS	
PNI								
<48	223	13.1	12.6‐13.6	.427	NS	NS	NS	NS
≥48	196	13.5	12.6‐14.4		NS	NS	NS	
CACI								
≤4	236	13.8	13.1‐14.5	.008	NS	NS	NS	NS
>4	183	12.5	11.9‐13.1		NS	NS	NS	
CA19‐9								
<200 U/mL	163	13.9	13.2‐14.6	.025	NS	NS	NS	NS
≥200 U/mL	256	12.5	11.9‐13.1		NS	NS	NS	
CA19‐9 response								
≥74 U/mL with response	193	13.8	13.1‐14.4	<.001	1			<.001
Remain <74 U/mL	92	14.8	14.0‐15.6		0.80	0.60‐1.06	−0.23	
≥74 U/mL with no response	134	9.3	8.1‐10.4		2.88	2.25‐3.70	1.06	
BED_10_								
≥60 Gy	225	14.7	14.3‐15.1	<.001	1			<.001
<60 Gy	194	10.8	10.0‐11.5		2.59	2.06‐3.26	0.95	

VAS, visual analog scale; BED_10_, biological effective dose, α/β=10; SIRI, systemic inflammation response index; PNI, prognostic nutritional index; CACI, Charlson age‐comorbidity index; NS, nonsignificant.

**Figure 1:**
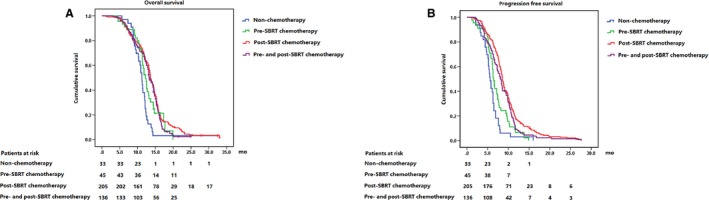
OS (A) and PFS (B) of patients with different combinations of treatment modality

Additionally, the median OS of patients with nonchemotherapy, induction chemotherapy, adjuvant chemotherapy and induction and adjuvant chemotherapy was 11.2 months (95% CI: 10.5‐11.8 months), 12.2 months (95% CI: 11.3‐13.0 months), 13.6 months (95% CI: 13.0‐14.1 months), and 13.3 months (95% CI: 12.4‐14.2 months) (Figure 1A). Patients with induction chemotherapy, adjuvant chemotherapy, or induction and adjuvant chemotherapy all had a longer OS than patients without chemotherapy due to systemic disease or poor performance status (nonchemotherapy vs induction chemotherapy: *P* = .018; nonchemotherapy vs adjuvant chemotherapy: *P* < .001; nonchemotherapy vs induction and adjuvant chemotherapy: *P* < .001), while no significant difference was found between these three multimodality (induction chemotherapy vs adjuvant chemotherapy: *P* = .107; induction chemotherapy vs induction and adjuvant chemotherapy: *P* = .450; adjuvant chemotherapy vs induction and adjuvant chemotherapy: *P* = .240).

### 3.4 Factors predictive of PFS

The median PFS was 8.2 months (95% CI: 7.9‐8.5 months), while 1‐year and 2‐year PFS rate was 21.5% and 14.1%, respectively. On univariate log‐rank comparisons, different combinations of treatment modality, CA19‐9 level, CA19‐9 response, and BED_10_ were associated with PFS (Table 3). On multivariate regression, a significant correlation was found between different combinations of treatment modality, CA19‐9 response, BED_10_, and PFS (Table 3).

**Table 3:** Univariate and multivariate analysis of factors associated with PFS

Variable	N = 419	Univariate, overall survival (mo)		*P* value (log‐rank)	Multivariate, hazard ratio			*P* value (Cox regression)
		Median	95% CI		HR	95% CI	*B*	
Age								
<65	191	8.1	7.7‐8.5	.243	NS	NS	NS	NS
≥65	228	7.8	7.3‐8.3		NS	NS	NS	
Smoking								
Absent	302	7.7	7.2‐8.1	.351	NS	NS	NS	NS
Present	117	8.4	8.0‐8.8		NS	NS	NS	
Diabetes mellitus								
Absent	322	8.1	7.7‐8.5	.168	NS	NS	NS	NS
Present	95	7.7	7.0‐8.4		NS	NS	NS	
VAS								
<3	158	7.5	6.4‐8.5	.807	NS	NS	NS	NS
≥3	261	8.1	13.1‐13.9		NS	NS	NS	
Weight loss								
<5 kg	221	7.9	7.3‐8.5	.369	NS	NS	NS	NS
≥5 kg	198	7.9	7.5‐8.3		NS	NS	NS	
Tumor diameter								
<4 cm	227	8.1	7.6‐8.6	.269	NS	NS	NS	NS
≥4 cm	192	7.8	7.3‐8.2		NS	NS	NS	
ECOG								
0	120	7.4	6.4‐8.4	.335	NS	NS	NS	
1	216	7.9	7.5‐8.3		NS	NS	NS	NS
2	83	8.5	7.9‐9.1		NS	NS	NS	
Treatment modality								
Nonchemotherapy	33	5.6	5.0‐6.2	<.001	1			<.001
Induction chemotherapy	45	6.4	6.0‐6.8		0.50	0.31‐0.79	−0.70	.003
Adjuvant chemotherapy	205	8.6	8.2‐9.0		0.28	0.19‐0.40	−1.29	<.001
Induction and adjuvant chemotherapy	136	8.1	7.4‐8.8		0.33	0.22‐0.49	−1.10	<.001
SIRI								
<1.0	230	8.2	13.3‐14.5	.077	NS	NS	NS	NS
≥1.0	189	7.6	11.8‐13.0		NS	NS	NS	
PNI								
<48	223	7.9	7.4‐8.4	.545	NS	NS	NS	NS
≥48	196	7.9	7.3‐8.5		NS	NS	NS	
CACI								
≤4	236	8.2	7.7‐8.7	.074	NS	NS	NS	NS
>4	183	7.8	7.3‐8.3		NS	NS	NS	
CA19‐9								
<200 U/mL	163	8.4	8.0‐8.8	.043	NS	NS	NS	NS
≥200 U/mL	256	7.6	7.1‐8.1		NS	NS	NS	
CA19‐9 response								
≥74 U/mL with response	193	8.5	8.1‐8.9	<.001	1			<.001
Remain <74 U/mL	92	9.5	8.4‐10.6		0.68	0.53‐0.88	−0.38	
≥74 U/mL with no response	134	5.6	5.0‐6.2		1.85	1.47‐2.32	0.61	
BED_10_								
≥60 Gy	225	9.8	9.1‐10.5	<.001	1			<.001
<60 Gy	194	6.2	5.7‐6.7		2.73	2.22‐3.36	1.00	

VAS, visual analogue scale; BED_10_, biological effective dose, α/β=10; SIRI, systemic inflammation response index; PNI, prognostic nutritional index; CACI, Charlson age‐comorbidity index; NS, nonsignificant.

Furthermore, the median PFS of patients with nonchemotherapy, induction chemotherapy, adjuvant chemotherapy, and induction and adjuvant chemotherapy was 5.6 months (95% CI: 5.0‐6.2 months), 6.4 months (95% CI: 6.0‐6.8 months), 8.6 months (95% CI: 8.2‐9.0 months), and 8.1 months (95% CI: 7.4‐8.8 months) (Figure 1B). In details, longer PFS was found in patients with adjuvant chemotherapy and induction and adjuvant chemotherapy (nonchemotherapy vs induction chemotherapy: *P* = .070; nonchemotherapy vs adjuvant chemotherapy: *P* < .001; nonchemotherapy vs induction and adjuvant chemotherapy: *P* < .001) (induction chemotherapy vs adjuvant chemotherapy: *P* < .001; induction chemotherapy vs induction and adjuvant chemotherapy: *P* = .034). Furthermore, it was indicated a marginal PFS benefit of adjuvant chemotherapy compared to induction and adjuvant chemotherapy (adjuvant chemotherapy vs induction and adjuvant chemotherapy: *P* = .048).

Though higher BED_10_ indicated better survival, there might be the potential impact of patients’ performance status and tumor diameters on the decision of prescription dose. Hence, further analysis was performed. No significant difference was found between ECOG and radiation doses (Table S1, *P* = .578) and between tumor diameters and prescription doses (Table S2, *P* = .860).

### 3.5 Adjusted survival benefit of adjuvant chemotherapy and induction and adjuvant chemotherapy

**Figure 2:**
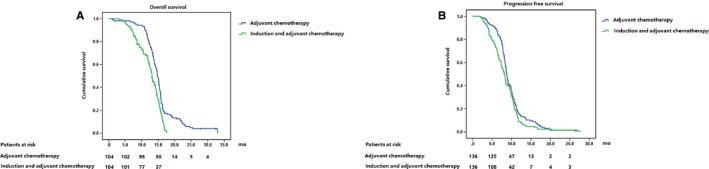
OS (A) and PFS (B) of patients with adjuvant and induction and adjuvant chemotherapy after adjustment

Regarding the survival benefit of adjuvant chemotherapy and induction and adjuvant chemotherapy, propensity score‐matched analysis was utilized for adjustment based on the previous factors predictive of OS and PFS. The median OS of patients with adjuvant chemotherapy and induction and adjuvant chemotherapy was 14.7 months (95% CI: 14.2‐15.2 months) and 13.1 months (95% CI: 12.3‐13.9 months) (*P* < .001) (Figure 2A). The median PFS of patients receiving adjuvant chemotherapy and induction and adjuvant chemotherapy was 8.8 months (95% CI: 8.4‐9.2 months) and 8.1 months (95% CI: 7.4‐8.8 months) (*P* = .053) (Figure 2B).

### 3.6 Tolerability of SBRT and chemotherapy

Regarding acute radiation‐induced toxicity, 146 patients had grade 1‐2 abdominal pain or nausea and vomiting. There was no grade 3 or more acute radiation‐induced adverse effects. Only 1 patient experienced grade 3 late toxicity event of duodenitis. Additionally, among patients receiving induction chemotherapy, 4 patients (4/45, 8.9%) and 5 patients (5/45, 11.1%) had grade 3 neutropenia and abdominal pain or nausea, respectively. In terms of adjuvant chemotherapy, 24 patients (24/205, 11.7%) experienced grade 3 neutropenia or leukopenia, while grade 3 abdominal pain or nausea and vomiting were found in 21 patients (21/205, 10.2%).

## 4 DISCUSSION

Optimal treatment modality for LAPC still remained controversial. Although combinations of radiotherapy and chemotherapy or other targeted therapy have been employed in practice, best combinations of treatment regimens and durations have yet to be reached a consensus. Less emphasis was placed on treatment strategies, especially the combination of SBRT and chemotherapy for LAPC. Only the combination of conventional radiotherapy and chemotherapy were explored.

In the previous studies, concurrent chemoradiotherapy and chemotherapy have long been an issue. In GERCOR,^36^ patients initially received gemcitabine‐based chemotherapy and were randomly enrolled in following chemoradiotherapy and maintenance chemotherapy. The OS and PFS were longer in patients with chemotherapy followed by chemoradiotherapy. While in the LAP07,^9^ there was no significant difference in overall survival between these two modalities with similar regimens compared with the previous one, but chemoradiation has resulted in an increase in PFS. In a retrospective study, Huang et al^37^ also compared chemoradiotherapy with or without induction chemotherapy vs chemotherapy alone. Results were in favor of first‐line chemotherapy with following chemoradiotherapy. Nevertheless, intensive induction schedule of chemoradiotherapy with sequential chemotherapy was proved more toxic and less effective than chemotherapy alone.^12^ Hence, upfront chemotherapy followed by chemoradiotherapy may be a better option.

Although it was indicated that no significant difference in overall survival with chemoradiotherapy compared with chemotherapy alone in LAP07,^9^ this study only investigated the impacts of induction chemotherapy with concurrent chemoradiotherapy, and the technique of radiotherapy was three‐dimensional conformal radiation therapy. Therefore, the treatment strategy and prognostic values may be different when SBRT was substituted for conventional radiotherapy. In the present study, a major OS benefit was shown in patients with induction, or adjuvant and induction and adjuvant chemotherapy compared with those with nonchemotherapy, but no difference was found between these three strategies. Besides, adjuvant and induction and adjuvant chemotherapy increased PFS compared with induction chemotherapy and nonchemotherapy. In addition, it was implicated a probable better prognosis in adjuvant chemotherapy than induction and adjuvant chemotherapy after adjustment. The underlying reason might be attributable to a somewhat better abscopal effect produced by SBRT than conventional radiotherapy,^38^, ^39^ rendering a synergic effect of SBRT and chemotherapy. However, the abscopal effect was rarely found in clinical practice, which may be ascribed to two contradictory mechanisms of radiation‐induced immune response, namely subversion and reinstatement of immunosurveillance.^40^

In our study, patients with lower ECOG tended to receive induction chemotherapy while stage III, lymph node involvement, and no radiation‐induced toxicity within 2‐3 weeks after SBRT correlated with the adjuvant chemotherapy. This might because patients with better performance status were more tolerant of chemotherapy and incidence of adverse effects of SBRT was probably lower than that of chemotherapy, which may indicate that upfront SBRT was more suitable for those with worse performance status. Additionally, more advanced stage required continuous intensive or consolidation chemotherapy after SBRT.

In our previous study, it was elucidated that patients receiving BED_10_ ≥60 Gy achieved better tumor response 6 months after SBRT though no correlation was found between the radiation dose and survival.^23^ However, it was shown in this study that BED_10_ ≥60 Gy associated with OS and PFS. Likewise, Krishnan et al^41^ also reported that BED_10_ >70 Gy was the predictor of OS. The effect of higher dose on survival may be independent of the biases because the inclusion criteria were stringent and confounders were assessed. However, the possibility of influence of intangible factors could not be precluded. Therefore, this should be further validated. The limitation of the study was nonrandomization. Though it was commonly accepted that chemoradiotherapy was the standard modality for LAPC, which was consistent with the finding that chemoradiation improved survival compared with radiotherapy alone, a prospective randomized study on comparison of different treatment sequences was required.

Generally, induction and adjuvant chemotherapy, especially adjuvant chemotherapy, may be beneficial for patients with LAPC. Upfront combined regimens of SBRT and chemotherapy were probably dependent of baseline ECOG and tumor stage, lymph node invasion, and toxicity after SBRT, respectively.

## CONFLICT OF INTEREST

The authors declared no conflict of interest.
