# Supplementary material for: Prospective analysis of different combined regimens of stereotactic body radiation therapy and chemotherapy for locally advanced pancreatic cancer
Source: Cancer Med. 2018 May 17;7(7):2913–24. doi: 10.1002/cam4.1553 (PMC6051203; doi:10.1002/cam4.1553)
Supplement: Supplementary file 1 [file CAM4-7-2913-s001.docx]

Supplementary table 1. Distribution of ECOG and BED_10_

|  | BED_10_≥60Gy (n) | BED_10_<60Gy (n) | P value |
| --- | --- | --- | --- |
| ECOG=0 | 56 | 64 | P=0.578 |
| ECOG=1 | 111 | 105 |  |
| ECOG=2 | 38 | 45 |  |

Supplementary table 2. Distribution of tumor diameters and BED_10_

|  | BED_10_≥60Gy (n) | BED_10_<60Gy (n) | P value |
| --- | --- | --- | --- |
| Tumor diameter <4cm | 121 | 106 | P=0.860 |
| Tumor diameter ≥4cm | 104 | 88 |  |
